# Process mining in mHealth data analysis

**DOI:** 10.1038/s41746-024-01297-0

**Published:** 2024-10-23

**Authors:** Michael Winter, Berthold Langguth, Winfried Schlee, Rüdiger Pryss

**Affiliations:** 1 Institute of Clinical Epidemiology and Biometry, University of Würzburg, Würzburg, Germany; 2 Institute of Medical Data Science, University Hospital of Würzburg, Würzburg, Germany; 3 Department of Psychiatry and Psychotherapy, University of Regensburg, Regensburg, Germany; 4 Eastern Switzerland University of Applied Sciences, St. Gallen, Switzerland

**Keywords:** Public health, Medical research

## Abstract

This perspective article explores how process mining can extract clinical insights from mobile health data and complement data-driven techniques like machine learning. Despite technological advances, challenges such as selection bias and the complex dynamics of health data require advanced approaches. Process mining focuses on analyzing temporal process patterns and provides complementary insights into health condition variability. The article highlights the potential of process mining for analyzing mHealth data and beyond.

## Introduction

This perspective article demonstrates the potential of process mining^1^ to provide clinical insights for smartphone health studies. Traditional clinical trials, mainly randomized controlled trials, are the gold standard^2^ for generating medical knowledge but have significant limitations. They are costly, time-consuming, sometimes ethically problematic (e.g., when patients are randomized to placebo treatment), and often need to represent the entire clinical population^3^. These studies assess the efficacy of specific interventions averaged over large samples, usually obscuring inter-individual differences and the temporal dynamics of individual treatment trajectories, which are also crucial for real-life clinical decisions^4^.

In recent years, intelligent telephone data collection from patients treated under real-life conditions has become increasingly important as an additional approach. This is mainly because ecological validity can be increased in this way.^5^. The data collected with these approaches have a fundamentally different structure than data from conventional clinical trials; e.g., data at various time points instead of data at “baseline” and “end of treatment”, high variability in the number of available data per person, but in return, you also get large samples and long observation periods. Approaches are therefore needed to exploit the full potential of these data sources. Using the TrackYourTinnitus mHealth project as an example, we want to show the advantages and limitations of process mining for the analysis of mHealth data.^6^. In our opinion, TrackYourTinnitus is particularly suitable because the project (1) has been running for over 10 years, (2) has generated representative EMA data, and (3) has already been examined in many publications. On this basis, an evaluation of process mining can be more comprehensive compared to previous analyses.

Our discussion begins with an overview of mHealth data collection, emphasizing the popularity of Patient-reported Outcome Measures (PROMs)^7^. Integrating PROMs with Ecological Momentary Assessments (EMAs) advances health data collection by mitigating observer and recall bias through real-time, field-based data capture^8^. EMAs ensure high ecological validity by repeatedly collecting data in real-world settings, typically via smartphone notifications^9^. Regular symptom reporting promotes self-reflection and enhances individuals’ understanding of their health, eventually improving health literacy^10^. Evidence from various studies, including cancer research^11^, underscores the effectiveness, scalability, and ease of use of EMAs and PROMs compared to traditional methods^12^.

Despite advancements, fully exploiting smartphone-based data for health research remains challenging due to technological overload and low adherence, often leading to selection bias^13^: Participants in long-term studies usually lose interest early, while those who are technologically adept or highly affected by the study’s focus contribute disproportionately more data. This can cause significant selection bias and unexpected subgroups within the study^14^. Innovative research and sophisticated data analysis strategies, such as Dynamic Structural Equation Modeling (DSEM) or Idiographic Machine Learning (IML), are increasingly used to address these issues^15,16^. Yet, the complexity and variability of health data often require additional approaches to complement the robustness and interpretability of analyses.

This brings us to the core of our perspective article: the potential of process mining in mHealth research, demonstrated by our TrackYourTinnitus project^6^. Our analysis suggests that process mining^17^, focusing on data sequences, temporal patterns, and state transitions, provides additional insights into the dynamics of health conditions, as seen in daily tinnitus symptom reports^6,10^. Process mining enables retrospective analysis to highlight process dynamics through visual process maps, offering improved clarity and understanding by encapsulating process state transitions, such as symptom changes, enhancing the interpretability and explainability of the results.

**Fig. 1:**
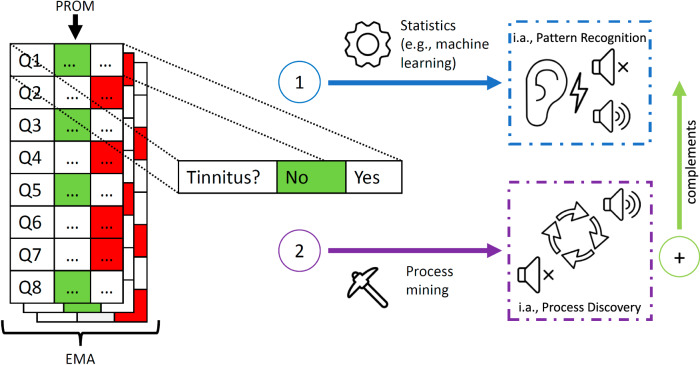
Data analysis techniques (i.e., machine learning, process mining) for EMA on the first question of the TrackYourTinnitus (TYT) daily 8-question questionnaire. This figure illustrates the data analysis techniques used for the Ecological Momentary Assessment (EMA) questions (i.e., first question (Q) of TYT daily 8-question questionnaire) in the TYT project. The first technique, 1) machine learning, is employed, among other things for pattern recognition, while 2) process mining is used for process discovery, complementing existing data-driven techniques. This comparison helps in understanding the dynamics of tinnitus symptom reports, showcasing how process mining can reveal - inter alia - process state transitions and symptom changes over time.

The complementary roles of process mining and existing data-driven techniques, such as machine learning, are illustrated using mHealth data from TrackYourTinnitus (TYT) in Fig. 1. Since 2013, TYT has tracked participants’ tinnitus variations through daily EMAs and PROMs^6,10^. Figure 1 focuses on the first question of the daily 8-question questionnaire, which asks whether participants currently perceive tinnitus when asked (a simple yes/no answer). Machine learning has been extensively used to predict responses to this question based on the other seven questions (e.g.,^14,18^). While the applied data-driven techniques are crucial for - inter alia - pattern recognition, process mining complements and enhances these capabilities as an additional approach for analyzing the dynamic nature of participant responses over time. By examining the temporal sequences and patterns of reactions, process mining predicts when participants report tinnitus, the frequency of these responses, and their evolution (i.e., no tinnitus (0) or tinnitus (1); see Fig. 1), complementing other techniques for a more comprehensive data analysis.

By exploring the application of process mining to analyze the EMA data of the TrackYourTinnitus project, we aim to illustrate the potential of process mining in mHealth and invite further exploration of its broad applicability in various other health datasets.

## Process Mining in a Nutshell for EMA Data

Process mining is a data analytics technology that bridges traditional business process management and data mining^17^. It analyzes event logs from information systems within organizations (e.g., companies^19^) to provide insights into operational processes by visualizing the temporal flow of operations. These insights help organizations to discover, monitor, and optimize processes to ensure efficiency and effectiveness. Process mining reveals how processes are executed^20^, revealing weaknesses and barriers. In healthcare, it may further improve the understanding and management of diseases by identifying and analyzing relevant processes^21^.

**Fig. 2:**
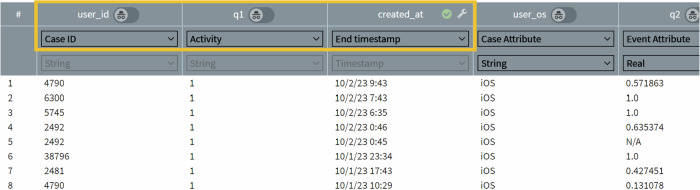
Definition of Ecological Momentary Assessment (EMA) data for process mining. This figure demonstrates the structure definition of EMA TYT data for the first question (i.e., tinnitus perception with no (0)/yes (1) of the TYT daily 8-question questionnaire for process mining. In this example (see orange area), the user_id functions as the unique case identifier, responses to the first question are represented as activities, and the record creation timestamp offers the essential chronological sequence. Additional elements in the dataset can be interpreted from different perspectives, such as case attributes or event attributes.

**Fig. 3:**
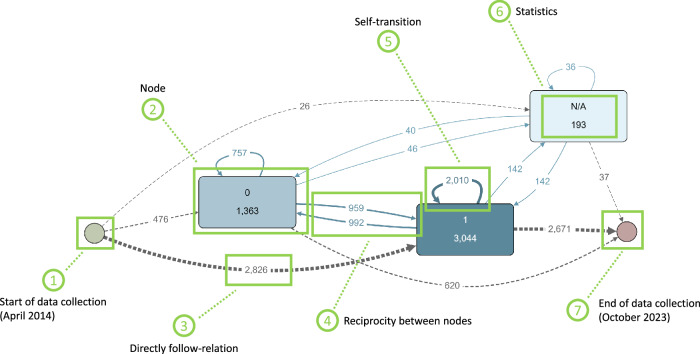
Ecological Momentary Assessment (EMA) data from TYT regarding tinnitus perception visualized as behavioral process map. This figure presents EMA data from TYT regarding tinnitus perception (i.e., 0 = no, 1 = yes, N/A = not available) visualized as behavioral process map. It highlights key events and transitions in participants' tinnitus perception over time. The process map includes events (1) & (7), nodes (2), dependencies (3), (4), & (5), and statistics (6).

Event logs are central to process mining, generated automatically by various IT systems, and contain detailed information about process activities, including timestamps, activity names, resources, and actors^22^. By analyzing this data, process mining tools reconstruct the actual process steps, allowing companies to visualize processes and identify bottlenecks, deviations, and inefficiencies^23^. Thereby, IDs and corresponding activities with related timestamps extracted from an event log are the basic requirements for analyzing and visualizing data with process mining^17^. The IDs help in identifying unique cases, while the activities and timestamps provide the necessary information to track the sequence and timing of events. Similarly, mHealth data from sensors (e.g., heart rate) or EMAs (i.e., questionnaire data), which can be structured as event logs, are ideal candidates for analysis using process mining techniques. Figure 2 illustrates how EMA data from TYT are structured for process mining. In this example (see Fig. 2; orange area), the user_id serves as a unique case ID, the answers to the first question of the daily TYT questionnaire (i.e. tinnitus perception no (0)/yes (1)) are treated as activities, and the timestamp for the creation of the dataset provides the necessary chronological information. Additional elements in the dataset can be defined as different viewpoints, such as case or event attributes. Based on this information, process mining can generate detailed insights, such as behavioral process maps (see Fig. 3), which visualize patient journeys from various perspectives (e.g., state transitions reflecting symptoms, treatment course, medication).

There are three main techniques in process mining: Ⓐ discovery, Ⓑ conformance, and Ⓒ enhancement:

Ⓐ Discovery involves analyzing event logs without a pre-existing model to map and visualize actual processes. In business, this helps in documenting processes and updating outdated models. In healthcare, it can compare individual symptom reductions with expected treatment improvements and identifies time windows for clinical care improvement.
Ⓑ Conformance checking compares event log with existing process models to verify alignment. It identifies discrepancies between planned and actual processes in business, highlighting improvement areas. In healthcare, it can compare individual symptom reductions with expected treatment outcomes and indicates time windows for clinical care improvement.
Ⓒ During improvement, existing process models are updated using insights from process mining to optimize performance. In healthcare, this can help tailor treatment processes for greater effectiveness for individuals.

In general, process mining offers valuable benefits^24^. It brings transparency to processes by uncovering hidden inefficiencies and bottlenecks^25^, leading to a better understanding and evidence-based decision-making. Process mining supports continuous improvement with a clear baseline and constant monitoring^26^, enhancing compliance and governance by ensuring processes meet standards and regulations^27^.

Process mining enables a better understanding of health data and optimizes health and treatment dynamics in real-world settings. EMA data, collected in real-time, reflect individuals’ symptoms, behaviors, and environmental contexts^28^. Applying process mining to EMA data may uncover patterns and sequences in symptoms and behaviors, complementing traditional methods, and leading to an improved understanding of disease progression and treatment effects. This technique may reveal how symptoms or behaviors are triggered by contextual factors or emotional states, providing insights into human behavior’s dynamic nature. Process mining can also support developing tailored interventions and optimizing their timing, contributing to more effective health, psychological, and behavioral interventions^29^.

## Process mining on EMA Data in practice: TrackYourTinnitus

TrackYourTinnitus (TYT) is a mHealth platform that uses EMAs to help individuals monitor and analyze their tinnitus in real-time via mobile apps. The app regularly prompts users to report their current tinnitus experience and tracks additional data such as environmental noise, daily activity, stress, and time of day. TYT collects data on daily activities and events related to tinnitus fluctuations, providing valuable insights for personal management and research. It also includes clinically relevant questionnaires like the Mini-Tinnitus Questionnaire (Mini-TQ) and the Tinnitus Sample Case History Questionnaire (TSCHQ) as Patient-reported Outcome Measures (PROMs)^6^.

In the following, we use TYT data to illustrate the applicability of process mining on EMA data by relying on the tool Apromore^30^. The data from 3,328 participants, who provided a total of 111,644 EMA-responses to the question of whether the tinnitus is currently perceived (1) or not (0), are shown in Fig. 3 as a behavioral process map reflecting the response behavior of the TYT participants.

In general, this retrospective data analysis complied with ethical guidelines and approvals in accordance with the Declaration of Helsinki. All users of TYT read and approved the provided informed consent. The approving board was the Ethics Committee of the University Clinic of Regensburg; No. 15-101-0204. Figure 3 illustrates the development of responses regarding current tinnitus experiences, categorized as present (1), not present (0), or missing data (N/A). Given that TYT EMA data includes millions of data points (especially unique patient IDs, activities, and timestamps) collected over more than 10 years, it is particularly suitable for process mining in this context. Missing data (N/A) can be included in the process map for consideration regarding data quality. Initially, 2826 participants reported having tinnitus, 476 did not, and 26 had inaccurate responses, totaling 3,044 tinnitus sufferers. From April 2014 to October 2023, out of 3328 participants, 284 never perceived tinnitus in any assessment. Among those reporting tinnitus, 2010 consistently experienced it, while 992 reported changes between “experiencing tinnitus" and “not experiencing tinnitus". The figure shows that most participants perceive tinnitus, with fewer experiencing fluctuations between presence and absence than those with a permanent perception.

After outlining one aspect of the behavioral process map shown in Fig. 3, the following list provides a detailed explanation of each aspect:

① Start event: Each process map begins with an atomic start event, marking the initiation of a specific instance and the point at which all relationships begin. In our illustration (see Fig. 3), it represents the start of data collection in April 2014. In other contexts, it could mark the beginning of clinical treatment, vaccination, behavioral change, or diet.
② Node: A node in a process map is a fundamental element that captures an instance of an action taken or a specific state. A node in Fig. 3 represents one possible response choice (i.e., tinnitus is present (= 1)).
③ Sequential order relation: These relations help understand the basic order between nodes, highlighting the intended sequence of steps or states and the dependencies between them. For instance, 2826 participants reported tinnitus perception directly after starting their data collection, as shown in Fig. 3. Although each patient’s start time varies, process mining summarizes these variations.
④ Reciprocity: Reciprocity refers to a bidirectional relationship where each node is both a predecessor and a successor of the other, indicating cyclical dependency. This means that the occurrence of one node likely leads to the occurrence of the other, and vice versa. In Fig. 3, 992 participants indicated tinnitus absence at a certain assessment, despite having perceived tinnitus in prior assessments.
⑤ Self-transition: A self-transition occurs when a node transitions back to itself without moving to a different node, indicating the same step or state is repeated consecutively. For example, Fig. 3 shows that 2,010 participants experienced tinnitus perception multiple times in succession. In a clinical context, this means the clinical state remains within the defined parameters of this state.
⑥ Statistics: The characteristics of a node within a process map can provide insights about human behavioral responses. For example, the response frequency depicted in Fig. 3 provides information about the distribution of given answers regarding tinnitus perception.
⑦ End event: Similar to ①, each process map concludes with an atomic end event, where all existing relations merge, indicating that all required steps or states have been covered, reaching the intended outcome. In Fig. 3, this marks the end of data collection in October 2023. In other contexts, the end event could signify hospital discharge or the end of clinical treatment.

**Fig. 4:**
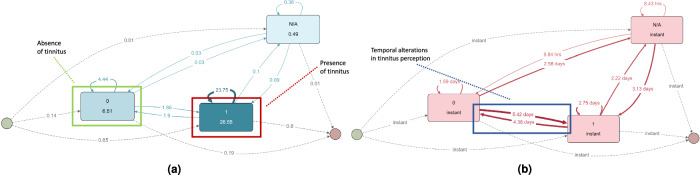
Other perspectives depicted in process maps. **a** Other perspectives depicted in process maps. This figure shows the average number of times participants reported the presence and absence of tinnitus over the course of the TrackYourTinnitus study. Participants reported tinnitus presence (1) an average of 27 times (see red area) and tinnitus absence (0) an average of 7 times (see green area). This data highlights the variability in tinnitus perception among participants. **b** Other perspectives depicted in process maps. This figure illustrates the time intervals between participants' responses regarding tinnitus perception. It highlights the changes between perceiving and not perceiving tinnitus over a period of days. For example, it appears that changes between tinnitus presence and absence typically occur after 4 or 5 days (see blue area). These temporal insights are crucial for understanding the cyclic nature of tinnitus and can help in tailoring intervention strategies to manage symptoms more effectively.

Process mining techniques allow analyzing EMA data, as illustrated in Fig. 4a, b, which present the process of Fig. 3, from different perspectives: Fig. 4 (a) shows patterns in mean response behavior, revealing that individuals reported tinnitus presence 27 times and absence 7 times, indicating variability in tinnitus perception. This granularity allows for targeted health strategies tailored to individual needs. Figure 4b, in turn, highlights temporal aspects, showing time intervals between responses, such as changes between tinnitus presence and absence occurring after 4 or 5 days. These temporal insights help understand the cyclicality of conditions or intervention effects over time. Additionally, process mining provides statistics on state frequency, duration, transition probabilities, and conformance checking, offering a comprehensive understanding of data and revealing process dynamics, bottlenecks, and patterns for more effective interventions and personalized treatments.

**Fig. 5:**
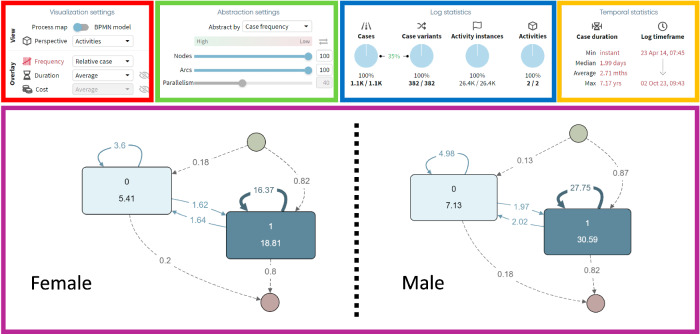
Application interface of process discovery regarding tinnitus perception for female and male TrackYourTinnitus (TYT) users. This figure depicts the application interface of process mining, while analyzing the tinnitus perception between female (i.e., left) and male (i.e., right) users of TYT. The red area represents visualization settings, in which different perspectives and statistics can be chosen (e.g., show different questions from TYT or other frequencies). The green area shows abstraction setting offering changes in the level of granularity for activities and arcs (e.g., show only 50 % of all activities, sorted by low case frequency), whereas the blue area contains specific log information such as number of cases and case variants. Temporal information (e.g., log time frame) are represented in the yellow area. Finally, the purple area shows the behavioral process maps, based on relative frequency, regarding tinnitus perception representing common user journeys between female and male users of TYT.

To further illustrate these concepts, the following case example highlights the applicability of process mining in EMA analyses. Specifically, it filters tinnitus perception based on sex (i.e., female & male). Figure 5 presents the application interfaces of process discovery showing the average frequency (i.e., average number of times a condition or transition occurs per user) of tinnitus perception in a behavioral process map for female (left) users, with the superimposed map for male (right) ones (e.g., on average, each female user experiences 18.81 tinnitus episodes, compared to 30.59 episodes in male users). We want to highlight a key advantage of process mining: its ability to visualize state transitions. For instance, the loop in Activity 1 for male participants, with an average frequency of 27.75, reveals that when males experience tinnitus, they are likely to encounter it repeatedly within a single case. Various settings are offered in the interface to analyze the data. Different perspectives (e.g., other questions from the TYT daily 8-question questionnaire) and statistics (e.g., frequencies) can be shown in visualization (i.e., red area). The level of granularity can be adapted in abstraction (i.e., green area). Log statistics (i.e., blue area) provide information about, for example, cases (i.e., individual users) and variants (i.e., common user journeys). Temporal statistics (i.e., yellow area) present specific time-dependent information. Finally, the behavioral process maps (i.e., purple area) depict the most common user journeys from the chosen perspective.

Based on these views, different operations can now be used to analyze the EMA data. For example, the definition of filters such as attribute (e.g., show only activities related to tinnitus perception), timeframe (e.g., process map should reflect only activities occurring during the morning), performance (e.g., identify users with high incidence of tinnitus), or path filters (e.g., show only state transitions from tinnitus non-perception to perception). These operations provide a robust framework for customized analysis of EMA data, enhancing insights and decision-making. Moreover, changes in visualization (e.g., view) or abstraction (e.g., level of granularity) enable the analysis of the behavioral process maps from different perspectives.

**Fig. 6:**
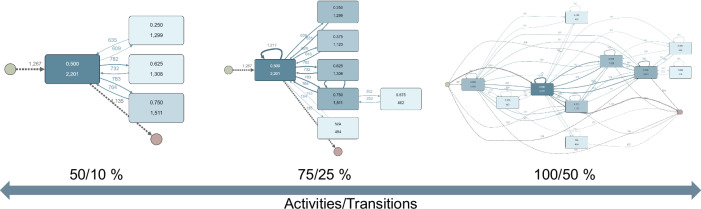
Dynamic real-time change of the level of granularity in process maps. This figure depicts how varying granularity levels in the process map, based on specific criteria like the most common patient journey, can impact the analysis of another question within the TrackYourTinnitus (TYT) data. It shows different granular perspectives by illustrating 50, 75, and 100 % of all activities, as well as 10, 25, and 50 % of all transitions. This approach highlights both the inherent variability and complexity within the data and enables a more targeted examination of the most significant or frequent paths by adjusting the visibility of complexity.

One of the strengths of process mining is its ability to make these adjustments dynamically and in real time. Figure 6 illustrates the impact of varying the granularity level in the process map according to specific definitions, such as the most common patient journey, based on another question within the TYT data. The figure shows different levels of granularity by depicting 50, 75, and 100 % of all activities, along with 10, 25, and 50 % of all transitions. On the one hand, this allows the high variability and complexity of such data to be revealed, and on the other hand, it enables a more targeted analysis of the most important or most frequent paths within the data, while selectively revealing or hiding the complexity.

Table 1 summarizes the results (i.e., state transitions; 1 = tinnitus, 0 = no tinnitus) for female and male based on case (i.e., number of users in which an activity occurs at least once), relative (i.e., percentage of users in which an activity occurs in relation to the total number of users), total (i.e., total number of occurrences of an activity), and average (i.e., average number of occurrences of an activity per user) frequency. The table shows tinnitus state transitions including 1090 female users with a total of 26,402 activity instances and 2199 male ones with a total of 82,945 activity instances. For example, regarding tinnitus perception, the table shows that out of 1090 female users, 991 (or 90.92 %) experienced tinnitus. Collectively, these women reported a total of 20,503 tinnitus episodes, with an average of 18.81 episodes per person. Based on Figs. 5 and 6, a comparison of tinnitus perception and related state transitions between female and male users reveals the following differences:

**Table 1:** Tinnitus perception analysis by sex

	Female				Male			
Number of cases	1090				2199			
Number of activity instances	26,402				82,945			
Case ∣ Relative ∣ Total ∣ Average - 1	991	90.92%	20,503	18.81	2026	92.13%	67,267	30.59
Case ∣ Relative ∣ Total ∣ Average - 0	480	44.04%	5899	5.41	864	39.29%	15,678	7.13
Case ∣ Relative ∣ Total ∣ Average - 1 to 1	663	60.83%	17,843	16.37	1326	60.30%	61,030	27.75
Case ∣ Relative ∣ Total ∣ Average - 0 to 0	271	24.86%	3920	3.60	477	21.69%	10,961	4.98
Case ∣ Relative ∣ Total ∣ Average - 1 to 0	343	31.47%	1788	1.64	633	28.79%	4433	2.02
Case ∣ Relative ∣ Total ∣ Average - 0 to 1	345	31.65%	1761	1.62	599	27.24%	4322	1.97

This table provides an analysis of tinnitus between female (i.e., total of 1,090 users with 26,402 activity instances) and male (i.e., total of 2,199 users with 82,945 activity instances) TYT users across various metrics, including the number of cases, activity instances, frequencies of (non-)tinnitus episodes, and related transitions (i.e., shifts from non-tinnitus (0) to tinnitus perception (1) and vice versa). The analysis includes several perspectives: case (i.e., the number of cases in which a particular condition or transition occurs at least once, helping to understand how common the condition is among the participants), relative (i.e., the percentage of cases in which a specific condition or transition occurs relative to the total number of cases, providing insight into the prevalence of the condition within each group), total (i.e., the overall number of times a particular condition or transition is recorded across all cases, highlighting the intensity or recurrence of the condition within the group), and average Frequency (i.e., the average number of times a condition or transition occurs per case, assessing the typical frequency of episodes experienced by each participant). For example, based on tinnitus perception, out of 2,199 men, 2,026 (or 92.13 %) experienced tinnitus. These men reported a total of 67,267 tinnitus episodes, with an average of 30.59 episodes per person.

**Female:** Exhibiting a slightly lower prevalence and intensity of tinnitus, with a total frequency of 20,503 and an average frequency of 18.81, 90.92 % of female participants experience tinnitus. However, they have higher variability in perception, transitioning more frequently between the states of tinnitus and no tinnitus, as reflected in a transition frequency of 1 to 0 of 31.47 % and a transition frequency of 0 to 1 of 31.65%. These values suggest a more dynamic symptomatology, with 60.83 % of cases showing stability in the tinnitus condition.

**Male:** Higher prevalence, intensity, and stability of tinnitus, with 92.13% of male participants experiencing tinnitus. This is reflected in the higher total frequency of 67,267 and an average frequency of 30.59. The lower frequency of transitions between tinnitus and no tinnitus is indicated by a transition frequency of 1 to 0 (i.e., tinnitus to no tinnitus) of 28.79% and a transition frequency of 0 to 1 (i.e., no tinnitus to tinnitus) of 27.24%. These values suggest that men are more likely to experience stable, persistent episodes of tinnitus, with 60.30% of cases showing stability in the tinnitus condition.

**Fig. 7:**
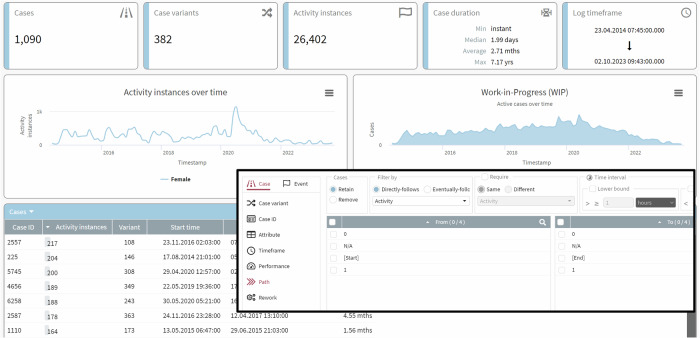
Process mining dashboard for detailed insights. This figure shows different statistics in a visual dashboard regarding tinnitus perception for female users of TrackYourTinnitus (TYT). The figure shows descriptive information concerning number of cases, variants, activity instances, and several temporal aspects. Regarding the latter, aspects (e.g., activity frequency over time) are presented, indicating variability in response behavior over time. User specific journeys are shown allowing for an intra- or inter-individual analysis. The figure highlights the application of filters (see black area) based on, for example, attributes (e.g., show only the occurrence of specific activities), temporal (e.g., filter out all activities, which appeared at night) or performance (e.g., retain all cases with a defined threshold for the frequency of activities) aspects.

In addition, such analyses are further enhanced by considering specific customizable dashboards, providing more in-depth information (see Fig. 7). Dashboards show details regarding descriptive statistics (e.g., number of cases and variants) and various temporal aspects (e.g., frequency of responses to the first question, highlighting changes in response patterns over time). Moreover, deeper insights can be gained for each user and their respective journey, whether considered from an intra- or inter-individual perspective. The application of filters enables sophisticated analyses (see Fig. 7; black area), such as observing changes in stress levels during transitions between tinnitus perception and non-perception, and vice versa.

The results obtained regarding differences in tinnitus perception between females and males based on the approach presented with process mining are supported by existing scientific studies^31,32^. A key advantage of process mining lies in its capacity to visualize and analyze dynamic patterns, enabling the tracing and mapping of pathways through which tinnitus symptoms emerge and fluctuate over time across different sexes. Since TYT captures further sociodemographic information (e.g., age, mobile system), the results indicate that sex differences in tinnitus perception are influenced by a complex interplay of biological, behavioral, and demographic factors^33^. By using the methods offered by process mining, these aspects could be further investigated to gain deeper insights into the underlying mechanisms that contribute to the observed sex differences in tinnitus perception.

Process mining offers therefore a valuable complement to existing data-driven techniques in the analysis of EMA data. Emphasizing temporal sequence relationships and enabling dynamic real-time adjustments, process mining, exemplified by the TrackYourTinnitus project, generates visual process maps that highlight the sequential and temporal patterns in patient-reported data, capturing the interplay of symptom variability and state transitions. This approach not only enhances the understanding of fluctuating symptoms, but also supports existing data-driven techniques like machine learning. This interplay of real-time adaptability and comprehensive analysis makes process mining a supportive tool for delivering improved personalized healthcare interventions.

## Clinical relevance

**Fig. 8:**
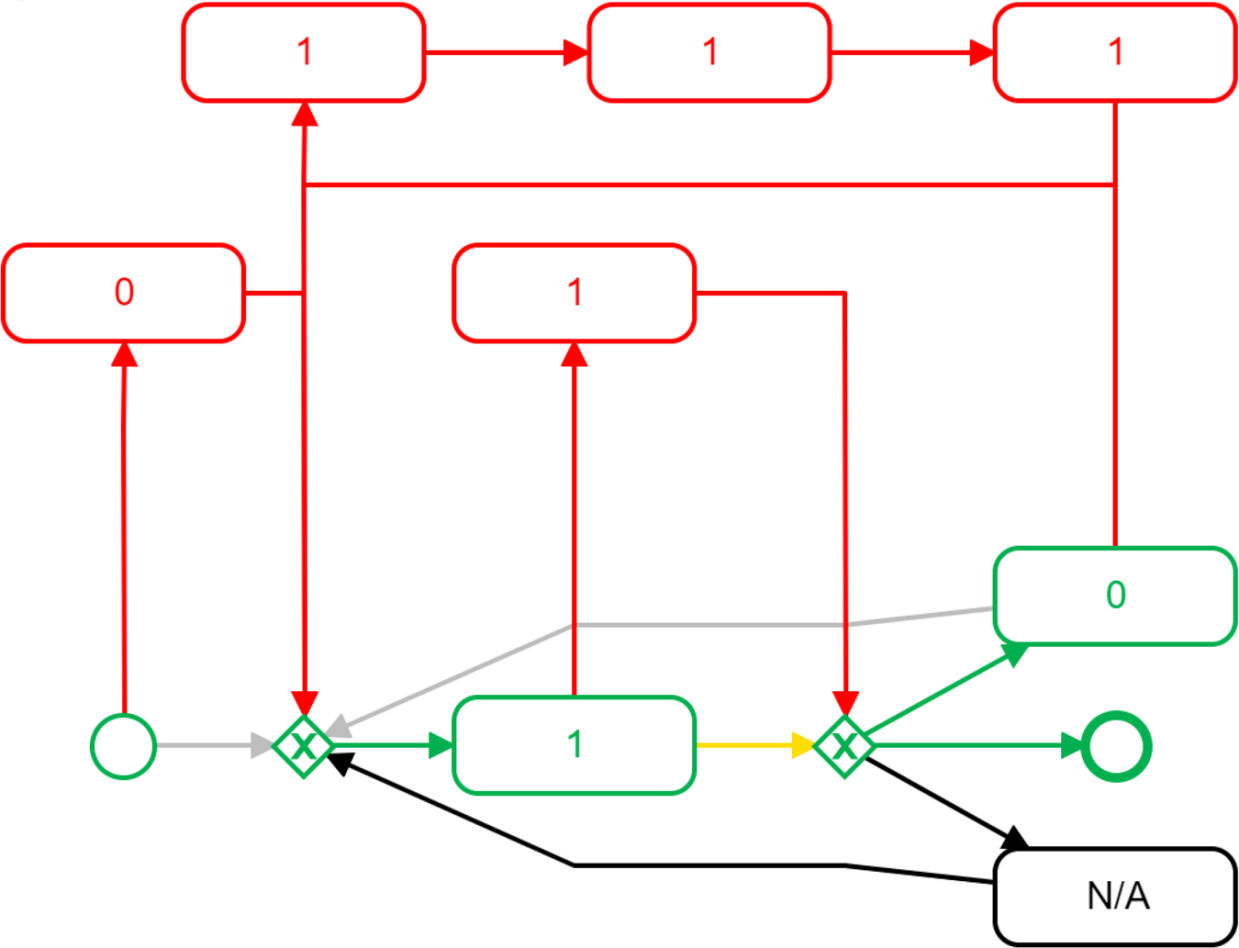
Conformance checking to identify deviations in patient journeys. This figure demonstrates the use of conformance checking to identify deviations in patient journeys from the overall sample. The conformance checking process compares individual patient data to the expected model, identifying consistent behaviors (green), deviations (red), movements (yellow), missing data (grey), and unutilized data (black). This analysis helps to highlight differences in patient behavior, uncovering potential issues such as incorrect treatments or external factors that may influence treatment outcomes. By identifying these patterns, healthcare providers can adjust treatment plans and offer more personalized interventions.

Before discussing the clinical significance, it’s important to mention the new insights from the process mining evaluation. The numerical data at state transitions offer an aggregate view of symptom changes, disregarding the temporal aspect of individual fluctuations, which may allow for more precise categorization of individuals. Thus, the clinical evaluation of EMA data through process mining holds potential for several reasons:

- Traditional analyses of EMA data involve longitudinal analyses of PROMs, which are often integrated into EMA. While PROMs reduce observer bias and improve health literacy, they have limitations, especially as they vary over time^5^. Therefore, process mining techniques complement traditional analyses of EMA data by offering an additional perspective. Process mining targets state transitions for the analysis of variability over time. By tracing transitions from “symptom perceived" to “symptom not perceived," process mining offers a supplementary perspective, improving understandability and interpretability of results.
- Given the dynamic nature of mental states^34^, an aggregated approach such as process mining on EMA data improves our understanding of these dynamics. This can be illustrated by the following two examples:
  1. Clinically, the transitions between “tinnitus perceived" and “tinnitus not perceived" are highly relevant. Aggregating these transitions allows for analyzing contextual factors that favor improvement (e.g., “tinnitus" to “no tinnitus" vs. “tinnitus" to “tinnitus") or worsening (e.g., “no tinnitus" to “tinnitus" vs. “no tinnitus" to “no tinnitus") of tinnitus.
  2. There is consensus that tinnitus varies significantly over time^6^. Analyzing state transitions with process mining can identify more homogeneous subgroups regarding fluctuations. For example, Fig. 8 shows how conformance checking can identify deviations of a patient compared to the overall sample. This method highlights similarities and differences in patient behavior within a defined subclass compared to the overall cohort, allowing the identification of improvement or worsening time points for further analysis of specific triggers (e.g., events). Conformance checking also reveals patterns of non-compliance, where patient behavior deviates from expected models, indicating potential issues like incorrect treatment or external factors. Identifying these patterns allows healthcare providers to adjust treatment plans and offer tailored interventions. Additionally, it helps monitor the efficacy of new protocols by comparing patient adherence and outcomes to standards, enabling continuous clinical improvement.
- A recent study found that while machine learning predictions using EMA data can sometimes face challenges, they still provide valuable insights. However, incorporating the most recent symptom occurrence as a predictor has proven particularly reliable^14^. In these scenarios, an aggregated overview of all symptom transitions enhances the analysis. This suggests that traditional methods are valuable, but integrating process mining offers additional information to better understand symptom dynamics.
- The best-established treatment for tinnitus is cognitive behavioral therapy (CBT), which helps patients focus away from tinnitus and reduce emotional involvement. This treatment usually takes several weeks to months and is effective for many, but not all, patients. Within the context of CBT, various techniques are available for behavioral modification^35^. Concomitant assessment of tinnitus perception and associated factors using EMAs, analyzed with process mining, could help to better understand:
  1. the dynamics of symptom improvement during a specific treatment (discovery)
  2. which contextual factors may facilitate effects of a specific treatment (discovery)
  3. in how many patient the treatment effects evolve as predicted and in how many patients the course of symptoms deviates from the expected treatment trajectory (conformance)
  4. targeted interventions for the states “no-improvement” or “worsening”; e.g., stopping, modifying, or switching treatments
- Given the complexity of analyzing EMA data due to several influencing factors^36^ and the proven importance of these factors^37,38^, process mining can be a complementary tool.

Overall, the findings enhance our understanding of tinnitus as a chronic stress-related disorder. Analyzing EMAs with process mining complements the insights obtained from traditional methods, offering an additional understanding of the dynamics of tinnitus symptoms, their variability, and influencing factors, both in the natural course of the condition and during specific therapeutic interventions.

## Discussion

Smartphone health data collection offers valuable information in real-world conditions and clinical studies, but its full potential is yet to be realized. The two main data collection methods are built-in sensors and EMAs of PROMs^13^. These methods produce data structurally different from traditional clinical datasets, necessitating innovative analysis approaches. Early studies reveal the need for additional approaches for this type of data to support existing ones^18,39^. Consequently, it is crucial to explore alternative methods, such as process mining, for analyzing smartphone-generated EMA data.

Addressing concerns about applying process mining to incomplete and subjective health data is crucial. Incomplete data poses a challenge that needs to be addressed by the application of process mining that otherwise leads to inaccurate and biased results^22^. For example, missing timestamps in patient records could obscure critical patterns in symptom progression or treatment efficacy, leading to incorrect conclusions^40^. To mitigate these risks, several approaches have been developed to address issues related to incompleteness in process mining. Abstractions simplify the event logs by focusing on higher-level activities, reducing the noise introduced by missing data^41^. Patterns allow the identification and reconstruction of likely missing events by analyzing common sequences of activities in complete logs^42^. Filtering techniques selectively remove or adjust incomplete cases, ensuring that the remaining data is of sufficient quality for reliable analysis^43^. Each of these methods plays a vital role in enhancing the robustness of process mining, particularly in complex and variable data environments typical of healthcare.

In our demonstrations (see Figs. 3 and 4) on the TYT data, missing data is represented as a separate activity in the process map. It ensures that missing data is not simply ignored or discarded, but rather treated as an informative event within the process. By doing so, we can identify specific points in time or particular sequences where data loss is more likely to occur, which indicate underlying issues such as patient disengagement, technological failures, or other external factors affecting data collection. Further, displaying missing data as a separate activity in the process map allows for improved analysis, helping to identify patterns or correlations with other events, such as specific PROMs or times of the day.

These findings highlight contextual factors that contribute to data gaps and inform strategies to improve patient adherence, adapt data collection protocols, or improve the technology used. Moreover, by visualizing missing data within the process map, we can assess its impact on the analysis, comparing patient paths with and without data gaps to better understand and ensure the reliability of the insights derived from process mining (e.g., conformance checking; see Fig. 8). Further, research showed that the application of appropriate methods complementing process mining with other data analysis methods (e.g., machine learning^44^) allows for the creation of a more comprehensive understanding, even when dealing with incomplete data. However, more research is needed on the handling of incomplete data in the context of process mining, particularly to refine these methods and ensure their effectiveness across various healthcare applications.

In healthcare, data must be handled with particular care due to unique challenges like high variability and data quality issues^45^. These challenges become even more pronounced when dealing with subjective data, such as that collected through EMAs, where variability and data quality issues are further compounded. Although the fundamentals of process mining rely on objective data, it can be adapted to effectively manage subjective reports by incorporating adjustments for variability and incompleteness, as demonstrated in this article. Given its subjective nature, EMA data can be effectively structured as event logs (see Fig. 2), capturing essential elements such as unique IDs (e.g., patient ID), activities (e.g., operations, treatments, states), and timestamps that reflect the temporal sequence of events^17^. This structuring enables the application of process mining techniques to subjective data. Importantly, while precise timestamps enhance the analysis, process mining remains effective as long as the temporal order of events is preserved (i.e., data records are stored in succession). This flexibility allows for meaningful pattern recognition and sequence analysis in EMA data, even when exact time points are not available^46^.

Process mining on subjective health data constitutes a method that maps how doctors typically gather information about patients^47^. Medical doctors ask whether symptoms have changed, when they have changed, and which contextual factors might have played a role (e.g., tinnitus increased on day X after a night with only 4 hours of sleep and loud music. It got better 6 hours later after taking medication Y). Thus, the questions of medical doctors focus on specific time points or periods characterized by state transitions (i.e., “low tinnitus loudness" to “high tinnitus loudness"; “high tinnitus loudness" to “low tinnitus loudness"). By focusing on these transitions, which can be enriched and compared with information from different methods (e.g., conformance checking (see Fig. 8), dashboards (see Fig. 7), and by aggregating data from large samples, process mining represents an additional analysis technique for examining subjective health data.

Even with minimal data, process mining can provide insights^48^. For instance, a single data point with an ID, activity, and timestamp is sufficient to create an initial process map. As datasets increase in size and complexity (e.g., having numerous activities), process mining uncovers comprehensive patterns, sequences, and trends, unveiling hidden complexities and providing valuable insights into underlying processes^49^. When EMA data is organized in this manner, subjective EMA data, such as tinnitus reports, can reveal significant insights. For example, conformance checking (see Fig. 8) allows for the identification of deviations in PROMs, highlighting issues such as incorrect treatments or the influence of external factors. Moreover, applying process mining to demographic data, such as sex (see Fig. 5, enhances the analysis of subjective symptom reports. Finally, by integrating process mining with other methods (e.g., machine learning^50^), a more robust analytical framework can be created that takes subjective and objective health data into account.

Combining process mining with existing data-driven techniques like Dynamic Structural Equation Modeling (DSEM) or Idiographic Machine Learning (IML) presents promising opportunities for future research, with the potential to enhance the analysis of temporal dynamics and causal relationships in mHealth data. DSEM addresses complex interdependencies among variables over time, capturing the dynamic nature of symptom fluctuations and their interactions within an individual’s daily life^51^. In turn, IML focuses on personalized, individual-level predictions, by tailoring models to the unique symptom trajectories and behavioral patterns of each patient, thereby improving the precision of interventions^52^. Process mining complements these approaches by uncovering sequential patterns and state transitions within the data, offering (visual) dynamic insights into the progression of symptoms and their triggers over time^53^. Together, these methods could contribute to a more comprehensive framework for improving predictive accuracy and clinical relevance in longitudinal mHealth studies, particularly in tracking how patient behaviors and symptoms evolve in response to treatments or interventions.

While process mining is valued for its rapid deployment and efficiency in evaluations^17^, its effective integration with approaches such as DSEM and IML requires further investigation. Research should concentrate on how process mining can be successfully combined with traditional data-driven techniques to tackle the unique challenges of subjective and incomplete EMA data, with a particular focus on evaluating the reliability, accuracy, and potential benefits of this integrated approach. Thereby, it is important to consider that the origins of process mining lie in other domains, such as business process management, However, as demonstrated in this article, process mining can also be effectively applied to EMA data. To fully harness its potential in this context, its methodologies may need further refinement to capture the full value for the analysis of subjective EMA data.

In summary, process mining provides an additional perspective by analyzing mHealth data process dynamics. This method complements existing techniques and can offer clinicians new insights by uncovering sequential data patterns and process states, giving a detailed view of patient journeys and treatment effectiveness. It identifies bottlenecks and inefficiencies in treatment processes, and its ability to be rapidly deployed without extensive parameter tuning makes it valuable for real-time process optimization. Additionally, process mining offers the following advantages:

1. **Holistic View of Patient Journeys**: Process mining captures the entire treatment process, from initial consultation to follow-up, providing a comprehensive view of patient care pathways that traditional methods might miss.
2. **Real-Time Process Monitoring and Optimization**: Process mining can be applied in real time to monitor healthcare processes as they occur, allowing for immediate identification and correction of inefficiencies.
3. **Powerful Filtering Capabilities:** Process mining allows for in-depth data analysis through filtering by timeframe, performance, and trajectory, enabling deeper insights into specific aspects of patient care and treatment processes. This helps uncover detailed trends and patterns that can inform more targeted and effective interventions.
4. **Adaptability to Individualized Care**: The technique supports the development of personalized treatment strategies by identifying patterns and transitions specific to individual patients, enhancing the efficacy of interventions.
5. **Complementary to Other Analytical Methods**: When used alongside methods like DSEM and machine learning, process mining enriches the analysis by adding insights into the sequential and process-oriented aspects of health data.
6. **Actionable Insights for Continuous Improvement**: The ability to continuously monitor and analyze processes supports a culture of continuous improvement, helping healthcare organizations to anticipate and prevent problems before they impact patient care.

This perspective article demonstrated that process mining constitutes a candidate to complement existing data-driven techniques in the analysis of EMA data. Using TrackYourTinnitus as an example, we found differences in the average number of ratings with perceived tinnitus (26.55) and without tinnitus (6.51), as shown in Fig. 4a. Figure 4b illustrates the time differences in completing these assessments. Clinicians can use this data to evaluate if individuals match the averages or deviate, providing new insights. These findings can also be integrated as features in other data-driven techniques such as machine learning models. Overall, Figs. 3 and 4a, b highlight numerous transitions between perceived tinnitus (1) and unperceived tinnitus (0), offering a more detailed perspective. Whereas Figs. 5, 6, 7, and 8 illustrate the application of various analysis capabilities offered by process mining.

Process mining rapidly extracts insights from (large) datasets without extensive parameter adjustments or long processing times^17^. This capability offers new ways to gain insights, especially in the context of mHealth and broader health data. It provides an important additional approach to analyzing PROM and EMA data with direct clinical implications and utility.

## Data Availability

The datasets used and/or analysed during the current study available from the corresponding author on reasonable request.
